# RFamide peptides, the novel regulators of mammalian HPG axis: A review

**DOI:** 10.14202/vetworld.2021.1867-1873

**Published:** 2021-07-20

**Authors:** Smruti Smita Mohapatra, Joydip Mukherjee, Dipak Banerjee, Pradip Kumar Das, Prabal Ranjan Ghosh, Kinsuk Das

**Affiliations:** Department of Veterinary Physiology, Faculty of Veterinary and Animal Sciences, West Bengal University of Animal and Fishery Sciences, Kolkata, West Bengal, India

**Keywords:** hypothalamic–pituitary–gonadal axis, reproduction, RFamide-related peptides-3

## Abstract

The RFamide-related peptides (RFRPs) are the group of neuropeptides synthesized predominantly from the hypothalamus that negatively affects the hypothalamo-hypophyseal-gonadal (hypothalamic–pituitary–gonadal [HPG]) axis. These peptides are first identified in quail brains and emerged as the mammalian orthologs of avian gonadotropin inhibitory hormones. The RFRP-3 neurons in the hypothalamus are present in several mammalian species. The action of RFRP-3 is mediated through a G-protein-coupled receptor called OT7T022. The predominant role of RFRP-3 is the inhibition of HPG axis with several other effects such as the regulation of metabolic activity, stress regulation, controlling of non-sexual motivated behavior, and sexual photoperiodicity in concert with other neuropeptides such as kisspeptin, neuropeptide-Y (NPY), pro-opiomelanocortin, orexin, and melanin. RFamide peptides synthesized in the granulosa cells, interstitial cells, and seminiferous tubule regulate steroidogenesis and gametogenesis in the gonads. The present review is intended to provide the recent findings that explore the role of RFRP-3 in regulating HPG axis and its potential applications in the synchronization of reproduction and its therapeutic interventions to prevent stress-induced amenorrhea.

## Introduction

The hypothalamic–pituitary–gonadal (HPG) axis or gonadotropic axis consisting of the hypothalamus, pituitary gland, and gonads is the key regulator of reproduction in mammals. It operates through coordinated mechanisms of hypothalamic gonadotropin-releasing hormone (GnRH) and hypophyseal gonadotropins (follicle-stimulating hormone [FSH] and Luteinizing hormone [LH]) with peripherally produced gonadal hormones [1]. GnRH, the key operator secreted in two modes from the gonadotropic axis – pulsatile and surge [2]. GnRH pulse generator at the mediobasal hypothalamic area controls the pulsatile release of GnRH [3]. Pre-optic area GnRH surge generator is responsible for pre-ovulatory LH surge [4]. Hypothalamus receives feedback signals from gonads through gonadal hormones. Estradiol exerts both negative and positive feedback in a dose-dependent manner during different reproductive cycle stages in females [5] in contrast to testosterone which has only negative feedback over the GnRH release. However, surprisingly, GnRH neurons lack estradiol receptor (ER) and deletion of GnRH-specific ERb has no effect on the reproductive cycle in mice [6]. These observations have led researchers across the globe to find out the mechanism behind the control of HPG axis and several neuropeptides controlling GnRH release. Kisspeptin neurons [7] and RFamide-related peptides (RFRP)-3 neurons [8] have emerged as the key neuropeptides having a stimulatory and inhibitory effect on GnRH secretion, respectively. The kisspeptin neurons located in the pre-optic area and median eminence regulates both GnRH surge and pulsatile center, respectively. Kisspeptin-induced GnRH secretion is well documented in rat [9], mouse [10], sheep [11], cow [12], rhesus macaque [13], and human [14]. Kisspeptin induces the positive feedback of sex steroids on HPG axis in rodents [15]. The role of kisspeptin on metabolic regulation of reproductive cyclicity through leptin is also reported [16]. However, compared to kisspeptin, much less attentions have been paid to inhibitory neuropeptides and RFamide-related peptides which emerged as the promising areas of research in reproductive endocrinology in mammals.

## Discovery of Gonadotropin Inhibitory Hormones (GnIH)/RFRPs

Tsutsui *et al*. [17] identified a group of neuropeptides that inhibit pituitary gonadotropin *in vitro* pituitary cell culture of adult male quail. It was named GnIH due to its selective inhibitory property over gonadotropins. The receptors of the GnIH have also been identified in the pituitary of quails and *in vivo* experiments confirm that GnIH administration inhibits the expression of a and b subunits of FSH and LH [18]. The sequencing of GnIH revealed Ser-Ile-Lys-Pro-Ser-Ala-Tyr-Leu-Pro-Leu-Arg-Phe-NH2 sequence and due C-terminal LPXRFamide sequence (X=L or Q), so termed as LPXRFamide peptides. The five classes RFamide peptides in mammals are GnIH, neuropeptide FF (NPFF), prolactin-releasing peptide, kisspeptin (kiss1 and kiss2), and pyroglutamylated RFamide peptide/26RFamide peptide (QRFP/26RFa). RFRP-1, -2, and -3 in mammals are orthologous to avian GnIH [19], of which RFRP-3 is considered as the true homolog of GnIH concerning gonadotropin secretion in mammals [20]. The RFRP-1, -2, and -3 are encoded by NPVF gene in humans [21]. Of the three RFamide-related peptides, RFRP-1 and RFRP-3 are reported to be functionally active [21]. RFRP-1 and RFRP-3 have been isolated from cattle [12], human [22], and Siberian hamster [23].

## Localization of RFRP-3 Neurons in the Hypothalamus

There are huge variations among species regarding the localization of RFRP-3 neurons in the brain. The sexual variations in the localization patterns are also found in different species. Table-1 summarizes the localizations of RFRP-3 in different parts of the hypothalamus[23-37].

**Table-1:** Localization of RFRP-3 neurons and its projections in the hypothalamus in different species.

Species	Localization of RFRP-3 neurons	Neuronal projections of RFRP-3 neurons	Reference
Human	DMN	Infundibulum, ME and POA	[24]
Sheep	DMN and PVN	Arcuate nucleus (ARC), PVN, ventromedial hypothalamic nucleus, DMN, and lateral hypothalamic area	[25-27]
Mice	DMN, the solitary tract nucleus, and lateral superior olive	Reticular nucleus, spinal trigeminal nucleus, parabrachial nucleus, up to the dorsal horn of the spinal cord except ME	[28]
Siberian hamster (male)	AH area, pre-mammillary nucleus, and DMN	Arcuate nucleus central gray, amygdala, lateral septal nucleus, medial POA	[23]
Siberian hamster (female)	DMN	ME	[29]
Rhesus macaque (male)	Intermediate periventricular nucleus	PVN, POA, IPe, ME, ARC, and dorsal hypothalamic area	[24]
Rhesus macaque (female)	Intermediate periventricular nucleus together with PVN and DMN	POA and medial basal hypothalamus	[30]
Rats (either sex)	DMN, ARC, lateral hypothalamic area	Hippocampus, PVN, POA, AH, lateral hypothalamus	[31-33]
Rats (male)	DMN, dorsal tuberomammillary nucleus, DMH, and VMH	Amygdala, diencephalon, MPOA, PVN	[34]
Naked mole-rats	DMH, ARC	MPOA, SEP, PVN, Arc, and ME	[35]
Pigs	PVN, DMN	Lateral hypothalamus	[36]
Mare	PVN, DMN	-----	[37]

RFRP=RFamide-related peptides, POA=Pre-optic area, MPOA=Medial pre-optic area, DMN=Dorsomedial nucleus, PVN=Paraventricular nucleus, ME=Median eminence, ARC=Arcuate nucleus, AH=Anterior hypothalamic, DMH=Dorsomedial hypothalamus, VMH=Ventromedial hypothalamus

## RFRP-3 Receptors

Nguyen *et al*. [38] identified two receptors for NPFF – NPFF1R and NPFF2R under the G protein-coupled receptor (GPCR) family. Hinuma *et al*. [21] reported a specific RFRP receptor called OT7T022 which showed binding capabilities against synthetic hRFRP-1 and hRFRP-3 ligands. HLWAR77 receptor reported by Elshourbagy *et al*. [39] showed specificity for both neuropeptides AF and NPFF. The functional identity between OT7T022, NPFF1R, and a GPCR (GPR147) has been reported. HLWAR77 and NPFF2R have functional similarities with GPR74. GPR147/NPFFR1 is the receptor for GnIH/RFRP-3. GPR147 is reported to be coupled with G^a^_i_, an inhibitory G protein that suppresses cAMP activity [21]. Gouardères *et al*. [40] reported that GPR147 can bind G^a^_s_ or G^a^_q_ proteins which explain the diverse role of RFRP-3. GPR147 has been identified in hypothalamus, pituitary, gonads, and accessory reproductive organs in birds [41]. Extra neural localization of RFRP-3 has been reported in male and female gonads of mice, Syrian hamsters, and rhesus macaques [42]. In testes, the predominant sites of GPR147 expression are spermatocytes and spermatids [43]. GPR147 has been identified in granulosa cells, theca cells, and luteal cells depending on the stages of the reproductive cycle [43].

## Functions of RFRP-3 in Reproduction

### Regulation of GnRH secretion

RFRP-3 inhibits GnRH synthesis and secretion from the hypothalamus directly or indirectly in mammals. The RFRP-3 secreting neurons are situated in close apposition with RnRH secreting neurons along with substantial evidence of GPR147 expression in GnRH secreting cells and their projections. Intercerebroventricular (ICV) injection of RFRP-3 suppresses GnRH neuronal activity in female rats and mice [44]. *In vitro* cell culture studies confirmed the suppression of GnRH neuronal firing rates [19]. The indirect effect of RFRP-3 on GnRH neurons is mediated through kisspeptin. In mice, around 9-16% of kisspeptin neurons in the AVPV/RP3V area and 25% of kisspeptin neurons in the ARC area express GPR147 and 19-35% of kisspeptin neuron projections [45]. Decreased expression of kisspeptin mRNA in the hypothalamus upon intracerebroventricular administration of RFRP-3 has been reported in rats [44] and mice [46]. Wu *et al*. [47] reported that RFRP-3 could block glutamate transporter 2 in the GnRH neurons induced by kisspeptin.

### Regulation of LH secretion

Controversial results on the effect of RFRP-3 on pituitary LH secretion have been documented in different animal models. RFRP-3 decreases LH secretion in gonadectomized male and female rats intravenously [48]. Rizwan *et al*. [31] reported no change in the LH secretion on IV administration in ovariectomized rats. Acute RFRP-3 administration through ICV route reports no change in LH secretion in both gonadectomized male and female rats [48]. Chronic ICV injections of RFRP-3 cause a non-significant decrease in LH secretion and pulsatility in adult ovariectomized rats [49]. Administration of RFRP-3 through the intraperitoneal route has no effect on LH secretion in adult male or female mice [20]. Ubuka *et al*. [23] and Henningsen *et al*. [50] reported decreased LH secretion on acute ICV administration of RFRP-3, but it caused increased LH secretion in males under both long-day and short-day photoperiod. Decreased LH pulse has been reported upon IV administration of RFRP-3 in both intact and ovariectomized sheep [27], but no change in LH concentration is also reported [51]. No changes in the LH pulse secretion and amplitude on intravenous RFRP-3 administration were reported in mare [37] and gilt [36]. Intravenous administration of RFRP-3 in postmenopausal women causes decreased LH secretion [52]. Kadokawa *et al*. [12] reported decreased LH pulse frequency without altering the mean LH concentration on IV administration of RFRP-3 in male cattle. Blocking LH secretion by RFRP-3 can be mediated by three routes such as RFRP-3-kisspeptin-GnRH-LH, RFRP-3-GnRH-LH, and RFRP-3-LH as per the collective evidence.

### Effect of RFRP-3 on gonads

The paracrine action of RFRP-3 on the gonads is established. Bentley *et al*. [41] reported that GnIH is synthesized in the granulosa cells, interstitial cells, and seminiferous tubules in birds. McGuire *et al*. [53] reported inhibition of testosterone synthesis by GnIH in avian testicular cell culture. Species such as rats [54], mice [55], Syrian hamsters [56], sheep [57], pigs [58], primates [59], and human [55] are able to synthesize RFRP-3 in their gonads. The GPR 174 receptors of RFRP-3 have been identified in the oviduct, epididymis, and vas deferens of birds [41]. The localization of GPR-174 has been reported in the granulosa cell layer of pre-ovulatory follicles and corpus luteum in premenopausal women in which RFRP-3 inhibits gonadotropin-induced progesterone synthesis [55]. Singh *et al*. [42] identified RFRP-3 receptors in the granulosa cells of both healthy antral follicles during proestrus and estrus and in luteal cells during diestrus in mice establishing the role of RFRP-3 in follicular development. Zhao *et al*. [56] identified RFRP-3 and its receptors in spermatocytes and spermatids by immunohistochemistry and *in situ* hybridization and reported increased expression of RFRP-3 and GPR147 during late spermatocytes indicating the role of RFRP-3 in sperm maturation. Anjum *et al*. [60] studied the expression of GnIH in the testis of mice and correlated with serum testosterone levels from birth to senescence and found that RFRP-3 may cause pubertal activation of senescence in mice testis.

### Effect of RFRP-3 on sexual photoperiodism

A photoneuroendocrine system regulates sexual photoperiodism in mammals comprised of the photoreceptors in the retina, hypothalamus (suprachiasmatic nucleus), and the pineal gland. The pineal hormone melatonin released on photoperiodic cues is the key communicator between reproductive activity and seasons [61]. Higher melatonin production occurs at night; thus, nocturnal melatonin production is higher in seasons with short-day length (autumn/winter) than spring/summer [62]. The mechanism behind melatonin-induced sexual photoperiodism is not well. However, studies have shown that melatonin modulates some gene expressions in brain loci [63]. GnIH expression and release are directly regulated by melatonin acting on Mel1c receptors specifically expressed in GnIH neurons of quails [64]. In contrast to mammalian seasonal species, GnIH expression is increased by melatonin, and consequently, GnIH-ir expression is increased in short photoperiod as to long photoperiod [64]. *In vitro* studies show that GnIH release has a diurnal rhythm and is increased during nighttime in quail hypothalamic explants [65]. Bentley *et al*. [41] studied that elimination of pineal gland and reduced melatonin secretion markedly decreased GnIH expression in photoperiodic quail and song sparrows. This can be revived after the replacement of melatonin [64].

### Role of RFRP-3 on pre-ovulatory LH surge

Vasoactive intestinal peptide (VIP) and arginine vasopressin (AVP) are the two important neurotransmitters of the suprachiasmatic nucleus (SCN), which controls the daily rhythms GnRH secretion and the timing of the pre-ovulatory LH surge [5]. Russo *et al*. [66] reported that AVP and VIP releasing nerve fibers were in close apposition with RFRP-3 and around 10% RFRP-3 neurons expressed VPAC1 and VPAC2 receptors. He demonstrated that injection of VIP decreased RFRP-3 activity in the afternoon, not in the morning hence in a time-dependent manner. Lower expression of RFRP-3 coincides with LH surge in mice [45] and Syrian hamsters [50]. Simonneaux *et al*. [67] proposed a model on the role of RFRP-3 on pre-ovulatory LH surge where he stated that AVP and VIPergic neurons of SCN were the main regulators of the internal circadian clock in response to light output. SCN-VIP route controls GnRH neuronal activity either directly or through RFRP-3 neurons at DMH, whereas the SCN-AVP route stimulates GnRH activity through kisspeptin receive positive feedback from estradiol. These coordinated mechanisms are responsible for timely LH surges under the dark and light cycle.

### Effect of RFRP-3 on sexual maturation

RFRP-3 inhibits gonadotropin secretion in pre-pubertal animals. Sun *et al*. [68] demonstrated that the expression RFRP3/GPR147 mRNA in the hypothalamus is gradually decreased with puberty onset in rats. Xiang *et al*. [46] reported that RFRP-3 inhibits LH in estradiol dependent manner in pre-pubertal mice, not in adults. The differential expression of RFRP-3 neurons in the nucleus accumbens shells that regulates sexual motivation between sexually quiescent and active rats highlights the role of RFRP-3 on sexual maturity [35]. Han *et al*. [44] demonstrated that ICV injection of RFRP-3 delays puberty onset in female rats. Thorson *et al*. [36] reported the suppression of LH in ovariectomized prepubertal gilts by RFRP-3. These findings suggest the role of RFRP-3 to suppress HPG axis before puberty. Johnson and Fraley [69] reported that there are no alterations in the pubertal onset in RFRP-3 and GPR 147 knocked down models. Lima *et al*. [70] reported that RFRP-3 and GPR147 gene variants are not associated with GnRH-dependent pubertal disorders.

### Role of RFRP-3 in stress-induced reproductive suppression

Stress is reported to inhibit HPG axis and hence the reproductive function. The role of GnIH on stress-induced reproductive suppression was first reported by Calisi *et al*. in house sparrows [71]. RFRP-3 causes stress in chicken [65], zebra finches [72], mice [73], and rats [74]. Acute immobilization stress in mice causes a rapid increase in RFRP-3 gene expression and protein level coincides with higher glucocorticoid secretion [75]. Kaewwongse *et al*. [74] reported similar findings in rats on foot shock stress. McGuire *et al*. [76] reported that metabolic stress increased GnIH expression in ovaries of songbirds. Jaroslawska *et al*. [73] reported decreased RFRP-3 expression on the cold temperature in mice. The expression of glucocorticoid receptors in GnIH neurons in quails and corticosterone administration inhibits GnIH mRNA expression in quails [77]. Corticosterone induces upregulation of RFRP-3 in the testis of European starlings [36]. Direct action of glucocorticoids on RFRP-3 secreting neurons was established in rat hypothalamic cell culture [77]. The molecular mechanism of glucocorticoid-mediated RFRP-3 activations is confirmed after identifying glucocorticoid response elements in the RFRP-3 precursor coding region [78]. Stress-induced infertility is reported to be reversed after silencing of RFRP-3 in female rats [79].

### Role of RFRP-3 on feeding behavior and energetics

Optimum energy status is an essential factor for reproduction. Neurochemical systems regulate it. RFRP-3 increases feed intake in rodents [48], sheep [80], and primates [81]. The localization of RFRP-3 neurons in the DMH and PVN plays an important role in energy balance as these areas of the hypothalamus play a significant role in feeding [82]. Qi *et al*. [25] reported that RFRP-3 neurons are projected to NPY, pro-opiomelanocortin (POMC), orexin, and melanin-concentrating cells, key regulators of feeding, and energy status of animals. Administration of RFRP-3 through ICV route is reported to stimulate feed intake in male and female rats [48]. The RFRP-3 neurons in the PVN are reported to give inputs to one-third of cells secreting CRH and oxytocin cells which can potentially impact feeding behavior and energy status in animals [83]. Talbi *et al*. [84] reported that the suppression of reproductive activity, weight gain, and hibernation in female desert jerboas is associated with higher RFRP-3 expression and found that ICV injection of RFRP-3 causes a 4-fold increase in food-intake female jerboas along with a decrease in POMC and increase in NPY expression levels. After this observation, he proposed a model to describe the regulation of energy balance and feeding by RFRP-3. According to his model, the projections of RFRP-3 exert a negative effect on anorexigenic POMC neurons and a positive effect on orexigenic NPY neurons to increase feed intake in female desert jerboas.

## Conclusions and Future Perspectives

RFRP-3 is a pleiotropic neuropeptide that emerged as the regulator of the HPG axis and some critical reproductive events in mammals. It plays notable roles in stress-induced reproductive functions, sexual photoperiodism, and metabolic regulation. Localization, molecular characterization, mechanism of action, and pharmacological roles of RFRP-3 have been studied in several species during the past few decades. Due to the variability among species and sex, the precise role of RFRP-3 is yet to be determined. RRP-3 acts as a bridge between nutrition, stress, and reproduction and can be employed as therapeutic interventions in stress-induced infertility in mammals.

## Authors’ Contributions

DB: Conceived the idea. JM and SSM: Collected the literature and drafted the manuscript. PKD, PRG, and KD: Corrected the manuscript. All authors read and approved the final manuscript.
